# SARS-CoV-2/ACE2 Interaction Suppresses IRAK-M Expression and Promotes Pro-Inflammatory Cytokine Production in Macrophages

**DOI:** 10.3389/fimmu.2021.683800

**Published:** 2021-06-23

**Authors:** Ioanna Pantazi, Ahmed A. Al-Qahtani, Fatimah S Alhamlan, Hani Alothaid, Sabine Matou-Nasri, George Sourvinos, Eleni Vergadi, Christos Tsatsanis

**Affiliations:** ^1^ Laboratory of Clinical Chemistry, Medical School, University of Crete, Heraklion, Greece; ^2^ Department of Pediatrics, Medical School, University of Crete, Heraklion, Greece; ^3^ Department of Infection and Immunity, King Faisal Specialist Hospital and Research Center, Riyadh, Saudi Arabia; ^4^ Department of Microbiology and Immunology, College of Medicine, Alfaisal University, Riyadh, Saudi Arabia; ^5^ Department of Basic Sciences, Faculty of Applied Medical Sciences, Al-Baha University, Al-Baha, Saudi Arabia; ^6^ Cell and Gene Therapy Group, Medical Genomics Research Department, King Abdullah International Medical Research Center, Riyadh, Saudi Arabia; ^7^ Laboratory of Virology, Medical School, University of Crete, Heraklion, Greece; ^8^ Institute of Molecular Biology and Biotechnology, FORTH, Heraklion, Greece

**Keywords:** macrophages, ACE2, COVID-19, SARS-CoV-2, IRAK-M, inflammation, cytokines, IL-6

## Abstract

The major cause of death in SARS-CoV-2 infected patients is due to de-regulation of the innate immune system and development of cytokine storm. SARS-CoV-2 infects multiple cell types in the lung, including macrophages, by engagement of its spike (S) protein on angiotensin converting enzyme 2 (ACE2) receptor. ACE2 receptor initiates signals in macrophages that modulate their activation, including production of cytokines and chemokines. IL-1R-associated kinase (IRAK)-M is a central regulator of inflammatory responses regulating the magnitude of TLR responsiveness. Aim of the work was to investigate whether SARS-CoV-2 S protein-initiated signals modulate pro-inflammatory cytokine production in macrophages. For this purpose, we treated PMA-differentiated THP-1 human macrophages with SARS-CoV-2 S protein and measured the induction of inflammatory mediators including IL6, TNFα, IL8, CXCL5, and MIP1a. The results showed that SARS-CoV-2 S protein induced IL6, MIP1a and TNFα mRNA expression, while it had no effect on IL8 and CXCL5 mRNA levels. We further examined whether SARS-CoV-2 S protein altered the responsiveness of macrophages to TLR signals. Treatment of LPS-activated macrophages with SARS-CoV-2 S protein augmented IL6 and MIP1a mRNA, an effect that was evident at the protein level only for IL6. Similarly, treatment of PAM3csk4 stimulated macrophages with SARS-CoV-2 S protein resulted in increased mRNA of IL6, while TNFα and MIP1a were unaffected. The results were confirmed in primary human peripheral monocytic cells (PBMCs) and isolated CD14+ monocytes. Macrophage responsiveness to TLR ligands is regulated by IRAK-M, an inactive IRAK kinase isoform. Indeed, we found that SARS-CoV-2 S protein suppressed IRAK-M mRNA and protein expression both in THP1 macrophages and primary human PBMCs and CD14+ monocytes. Engagement of SARS-CoV-2 S protein with ACE2 results in internalization of ACE2 and suppression of its activity. Activation of ACE2 has been previously shown to induce anti-inflammatory responses in macrophages. Treatment of macrophages with the ACE2 activator DIZE suppressed the pro-inflammatory action of SARS-CoV-2. Our results demonstrated that SARS-CoV-2/ACE2 interaction rendered macrophages hyper-responsive to TLR signals, suppressed IRAK-M and promoted pro-inflammatory cytokine expression. Thus, activation of ACE2 may be a potential anti-inflammatory therapeutic strategy to eliminate the development of cytokine storm observed in COVID-19 patients.

## Introduction

Severe acute respiratory syndrome coronavirus 2 (SARS-CoV-2), a positive-sense single-stranded RNA virus, is the causative agent of the Coronavirus Disease 2019 (COVID-19), which rapidly developed in a global pandemic and resulted in major public health and economic complications. COVID-19 was first discovered in Wuhan, Hubei Province, China in December 2019 and as of March 2021, more than 116 million cases and 2.58 million deaths have been confirmed (1). SARS-CoV-2 has genetic similarities with other coronaviruses, such as SARS-CoV and Middle East Respiratory Syndrome Coronavirus (MERS-CoV), but it is significantly more contagious and can be easily spread *via* airborne transmission or contact with contaminated surfaces (2). The clinical manifestations of COVID-19 include mild upper respiratory tract symptoms such as fever, cough, fatigue, sputum production, shortness of breath, sore throat and headache (3), or more serious complications such as respiratory failure, acute respiratory distress syndrome (ARDS), heart failure, and septic shock (4). The most common diagnostic procedures include nucleic acid detection with real-time PCR and antibody detection against SARS-CoV-2 with rapid tests in respiratory tract samples or blood serum, respectively (5). Since there is no specific therapy for COVID-19 and therapeutic strategies are mainly supportive, emphasis has been given in prevention with the recently implemented vaccination along with the existing control measures (transportation quarantines, wearing medical masks etc).

SARS-CoV-2 genome encodes for proteins that contribute to viral replication and RNA synthesis, as well as for the structural proteins, spike (S), envelope (E), membrane (M), and nucleocapsid (N), that comprise the spherical virion (6). The SARS-CoV-2 S protein is responsible for cell entry after binding to its main cellular receptor angiotensin converting enzyme 2 (ACE2) (7–9). The S protein is a homotrimeric class I fusion protein consisting of a receptor-binding subunit S1 and a membrane-fusion subunit S2 (10–12). After receptor engagement by the receptor-binding domain (RBD) of S1 subunit, a plasma membrane-associated serine protease, transmembrane Serine Protease 2 (TMPRSS2), cleaves SARS-CoV-2 S protein at the S1/S2 site and promotes fusion of viral membrane with the host-cell membrane by the S2 subunit and subsequent release of the viral genome into the host cytoplasm (7, 13). Upon infection, the viral products activate various immune cells *via* pattern recognition receptors (PRRs) and produce a substantial amount of inflammatory cytokines and chemokines, resulting in the phenomenon of cytokine storm and a widespread lung inflammation. Patients with severe COVID-19 show high levels of inflammatory mediators, such as interleukin (IL)-2, IL-7, IL-10, granulocyte colony-stimulating factor (G-CSF), tumor necrosis factor (TNF)α, chemokine (C-X-C motif) ligand (CXCL)10, monocyte chemoattractant protein (MCP)1, macrophage inflammatory protein (MIP)1a and especially IL6 in serum (14), suggesting that the disease severity depends on cytokine storms that lead to Acute Respiratory Distress Syndrome (ARDS), and subsequently to multiorgan failure and death (15).

ACE2 is a single-pass type I membrane protein that is expressed on the surface of various cells including airway epithelial cells, monocytes and macrophages (16). ACE2 regulates important processes such as blood pressure and inflammation (16). The cell- surface exposed enzymatically active domain of ACE2 is responsible for the hydrolysis of angiotensin (Ang) II into angiotensin (1–7, 17, 18). SARS-CoV-2 infection induces the endocytosis of ACE2 receptor together with SARS-CoV-2 in host cells, thus increasing the serum levels of Ang II (17, 18). Ang II is a vasoconstrictor, which can also act as a pro-inflammatory cytokine *via* Ang II type 1 receptor (AT1R), promoting activation of the NF-κB pathway and IL6 production (19, 20). Therefore, the decreased availability of ACE2 with the resulting higher levels of Ang II, can increase inflammation and lung injury, suggesting a role in the ARDS development following SARS-CoV-2 infection (21). Clinical studies in mouse models with loss of ACE2 function, indicated increased release of pro-inflammatory chemokines, such as CXCL1, CXCL5, MIP2, TNFα, increased neutrophil infiltration and exaggerated lung inflammation and injury (22). In a different model of metabolic inflammation, that of apolipoprotein (Apo)E-/- mice, deletion of ACE2 resulted in hyper responsiveness of macrophages to LPS and production of TNFα, MCP-1, IL6, and matrix metalloproteinase (MMP)-9 (23). In the context of SARS-CoV, which utilizes the same receptor as SARS-CoV-2, the virus resulted in reduced ACE2 activity, increased IL8 expression and NFkB activation in macrophages (24), suggesting that interaction of S protein with ACE2 enhances inflammatory cytokine production. Moreover, our team previously demonstrated that another coronavirus, MERS-CoV, regulated macrophage responses *via* engagement of its S protein with its receptor DPP4 (25).

In the present study we investigated the impact of SARS-CoV-2/ACE2 interaction in cytokine production and modulation of macrophage responses to TLR signals and its effects on inflammatory regulators, such as IL1R-associated kinase (IRAK)-M and peroxisome proliferator-activated receptor gamma (PPARγ). We further, evaluated the role of the ACE2 activator Diminazene aceturate (DIZE), as a potential therapeutic strategy for restoring the local balance of the Renin Angiotensin System (RAS) and ameliorate the pro-inflammatory action of the virus, since it was previously reported to suppress inflammation by reducing pro-inflammatory cytokine production induced by LPS (26).

## Materials And Methods

### Cell Culture

THP-1 monocytic cell line was maintained in suspension in RPMI 1640 medium (Thermofisher Scientific, Waltham, USA), supplemented with 1% L-glutamine, 1% sodium pyruvate, 50 μM β-mercaptoethanol, 10% fetal bovine serum (FBS) and antibiotics (10,000 U/ml penicillin and 10 mg/ml Streptomycin). Differentiation of THP1 monocytes to macrophages was induced by 15 ng/ml phorbol 12-myristate 13-acetate (PMA) (Sigma-Aldrich, St Louis, USA) in THP-1 cells that were seeded in 24-well plates at a final density of 4×10^5^ cells/ml. Differentiated (adherent) THP- 1 macrophages were stimulated with different concentrations of SARS-CoV-2 Spike-Membrane Recombinant Fusion Protein (10, 20, 50, 100 ng/ml; TP701119, OriGene, Rockville, USA) in the presence or absence of the TLR ligands LPS (100ng/ml; Sigma-Aldrich, St Louis, USA) or PAM3csk4 (1μg/ml; Tocris, Bristol, UK) for 12 hours. In another set of experiments, THP-1 macrophages were pre-treated with the ACE2 activator Diminazene aceturate (10 μM; D7770, Sigma Aldrich, St Louis, USA) for 6 hours and then stimulated with 50ng/ml SARS-CoV-2 Spike-Membrane Recombinant Fusion Protein along with the TLR ligands (LPS, PAM3csk4). The effects of SARS-CoV-2 Spike protein along with TLR ligands were also investigated in PBMCs and CD14+ monocytes. Peripheral Blood Mononuclear Cells (PBMCs) were isolated from human peripheral blood of healthy donors by Ficoll density gradient centrifugation. Specifically, blood was diluted 1:1 with PBS and 10ml of diluted blood was overlayed on 5ml Ficoll reagent and centrifuged at 400g for 30 minutes at room temperature. PBMCs were removed from the buffy coats and washed in PBS. Separation and isolation of mononuclear cells from adult peripheral blood was performed under sterile conditions using Ficoll - Hypaque centrifugation (Lymphoprep StemCell Technologies, Oslo, Norway). Naïve monocytes (CD14^+high^CD16^-^, CD14^+high^CD16^+^ and CD14^+low^CD16^+^) were selected by immunomagnetic separation. Specifically, PBMCs were first stained with immunomagnetic beads (human PanMonocyte Isolation kit, Miltenyi Biotec, Gladbach Germany) and were collected *via* negative selection after passing through magnetic columns (Miltenyi Biotec, Gladbach, Germany). The purity of isolation was more than 90% as was confirmed by flow cytometry. Monocytes were seeded in a density of 3x10^5^ cells in 96-well plates and were cultured in RPMI medium enriched with 10% FBS and 1% Pen Strep.

### MTT Assay

Differentiated THP1 macrophages were seeded at a density of 7x10^4^ cells/well in a 96-well plate and treated with SARS-CoV-2 Spike-Membrane Recombinant Fusion Protein at concentrations of 10, 20, 50, 100 ng/ml, for 6, 12 and 24 hours. At each timepoint, 11μl of MTT reagent (5mg/ml) per 100 μl of cell culture medium was added, and 4 hours later medium was removed and 100 μl isopropanol/HCl was also added into the wells. Cells were incubated for 5 minutes in a shaker incubator and then OD at 594nm was measured in a Multiskan FC Microplate Photometer (Thermo Fisher).

### Enzyme-Linked Immunosorbent Assay (ELISA)

THP-1 macrophages, PBMCs or monocytes were stimulated with SARS-CoV-2 Spike-Membrane Recombinant Fusion Protein in the presence or absence of TLR ligands, and their supernatants were collected for cytokine quantification. Cytokine production of IL6, IL8, TNFα and MIP1a was determined by ELISA (BioLegend, SanDiego USA) as indicated by the manufacturer.

### Real-Time PCR

For the mRNA level detection of IL6, IL8, TNFα, MIP1a, CXCL5, IRAK-M and PPARγ, total RNA was extracted from THP-1 macrophages, PBMCs or monocytes using TRI Reagent (Sigma-Aldrich, St Louis, USA). Eight hundred nanogram of total RNA were used for cDNA synthesis (TAKARA, Primescript RT Reagent kit, Tokyo, Japan). Amplification was performed using KAPA SyBr^®^ Fast Universal qPCR kit (Kapa Biosystems, Cape Town, South Africa). Denaturation was carried out at 95°C for 10 seconds, annealing and extension at 60°C for 30 seconds for 40 cycles in a StepOnePlus™ Real-Time PCR System (Applied Biosystems, Foster City, CA, USA). Data analysis was accomplished using the ΔΔCT method and GAPDH was used as the housekeeping gene. The primer sequences used in this study, were the following: IL6: forward 5’ GTCAGGGGTGGTTATTGCAT 3’ and reverse 5’ AGTGAGGAACAAGCCAGAGC 3’; IL8: forward 5’ TGTGAAGGTGCAGTTTTGCC 3’ and reverse 5’ CACCCAGTTTTCCTTGGGGT 3’; TNFα: forward 5’ GCCCAGGCAGTCAGATCAT 3’ and reverse 5’ TATCTCTCAGCTCCACGCCA 3’; MIP1a: forward 5’ CCCGGTGTCATCTTCCTAACC 3’ and reverse 5’ GTAGCTGTGGAGGTCACACG 3’; CXCL5: forward 5’ ACAGACCACGCAAGGAGTTC 3’ and reverse 5’ TCTTCAGGGAGGCTACCACT 3’; IRAK-M: forward 5’ CACAACGTTCAACCATGCTC 3’ and reverse 5’ TGTTTACTGCTGCTGCTGGT 3’; PPARγ: forward 5’ GCTGGCCTCCTTGATGAATA 3’ and reverse 5’ TTGGGCTCCATAAAGTCACC 3’; GAPDH: forward 5’ GGAAGGTGAAGGTCGGAGTCA 3’ and reverse 5’ GTCATTGATGGCAACAATATCCACT 3’.

### Western Blot

For Western blot, cell lysates were harvested with RIPA lysis buffer and protein concentration was determined using the Pierce BCA Protein Assay. Proteins were separated on 8% polyacrylamide gel containing Sodium-Dodecyl Sulphate, and then transferred to nitrocellulose membrane. For the detection of IRAK-M, primary antibody (anti-IRAK-M, ab-8116 Abcam) was incubated with membrane overnight at 4^0^C, washed with PBST (0.1%Tween) and then incubated with HRP-conjugated secondary antibody for 1 hour at room temperature. Visualization of membranes was performed using the ECL system (Pierce) and a ChemiDocTM XRS+ (Bio-Rad).

### Flow Cytometry

Cell surface staining of blood mononuclear cells and isolated monocytes was carried out by incubation with FITC antihuman CD14 and APC anti human CD14 (BioLegend, San Diego, CA, US). Expression of protein levels of IRAK-M was determined by flow cytometry intracellular staining. THP-1 cells and PBMCs were fixed and permeabilized (Transcription Factor Staining Buffer Set, Thermo Fisher Scientific, Waltham, MA, US). Then, staining was carried out with anti-human IRAK-M rabbit polyclonal antibody (ab-8116, Abcam) and anti-rabbit APC conjugated secondary antibody (BioLegend, San Diego, CA, US). The proper isotype control was used as a control. The flow cytometry events were acquired in a FACS Calibur (BD Biosciences, San Jose, CA) and analyzed with the use of Summit v4.3 Software.

### Statistical Analysis

Comparison among groups was performed using One-Way-ANOVA, Mann – Whitney U test or the Kruskal - Wallis test with the Sidak or Dunn multiple comparison post-test were necessary. Data were depicted in box-and-whiskers or bars and plotted as mean ± SD or median (min, max). The GraphPad InStat Software (GraphPad v6.0, San Diego, CA, USA) was used for analysis. P value < 0.05 was considered statistical significant.

## Results

### SARS-CoV-2 S Protein Triggers Pro-Inflammatory Cytokine and Chemokine Expression in THP-1 Macrophages

**Figure 1:**
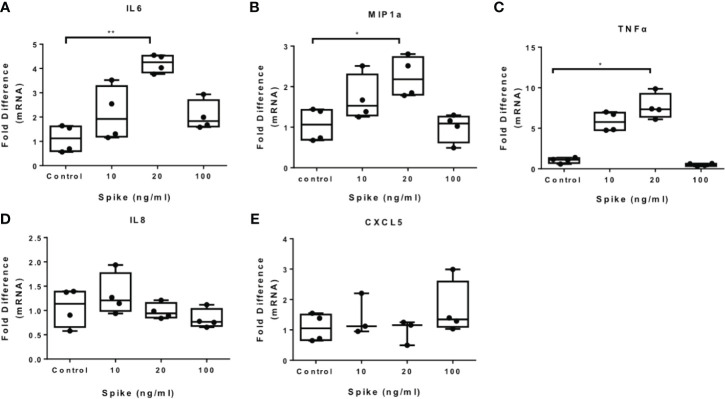
SARS-CoV-2 S protein induces pro-inflammatory cytokine and chemokine expression in THP-1 macrophages. THP-1 macrophages were treated with different concentrations of SARS-CoV-2 Spike-Membrane recombinant fusion protein (10, 20, 100 ng/ml) for 12 hours. **(A–E)** mRNA expression of IL6, MIP1a, TNFα, IL8 and CXCL5 was measured. Data are illustrated in box-and-whiskers and plotted as median (± min, max). Statistical analysis was performed with Kruskal - Wallis test. *p < 0.05, **p < 0.01.

To determine the immunomodulatory effect of SARS-CoV-2 -Spike/ACE2 receptor engagement, we stimulated THP-1 macrophages, derived from PMA treatment, with different concentrations of SARS-CoV-2 Spike-Membrane recombinant fusion protein and measured the induction of the inflammatory mediators IL6, TNFα, IL8, CXCL5, and MIP1a. We utilized a commercially available SARS-CoV-2 S protein raised in HEK293 cells, therefore no bacteria were involved, to avoid potential endotoxin contamination. The results showed that SARS-CoV-2 spike induced mRNA expression of IL6, MIP1a and TNFα (**Figures 1A–C**). No effect was observed for IL8 and CXCL5 (**Figures 1D, E**). The results suggest that SARS-CoV-2 S protein directly promotes pro-inflammatory cytokine and chemokine expression in macrophages. SARS-CoV-2 S protein did not affect macrophage survival (**Supplementary Figure 1**).

### SARS-CoV-2 S Protein Modulates TLR4 and TLR2 Responses in THP-1 Macrophages

**Figure 2:**
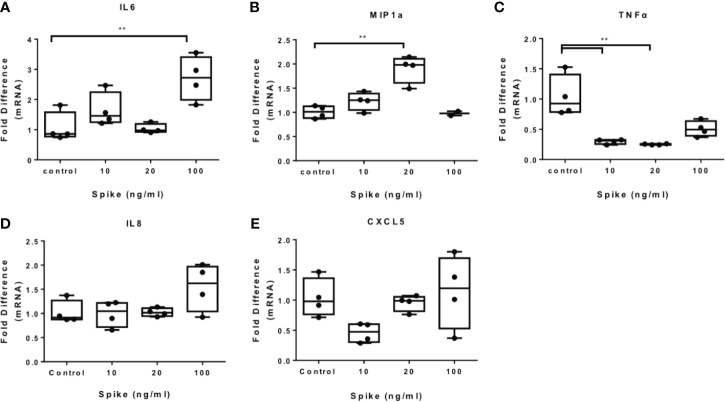
SARS-CoV-2 S protein increased cytokine expression in LPS-induced THP-1 macrophages. THP-1 macrophages were treated with various concentrations of SARS-CoV-2 Spike-Membrane recombinant fusion protein and LPS (100ng/ml) for 12 hours. **(A–E)** LPS-induced IL6, MIP1a, TNFα, IL8 and CXCL5 expression was measured. Data are illustrated in box-and-whiskers and plotted as median (± min, max). Statistical analysis was performed with Kruskal - Wallis test. *p < 0.05, **p < 0.01.

**Figure 3:**
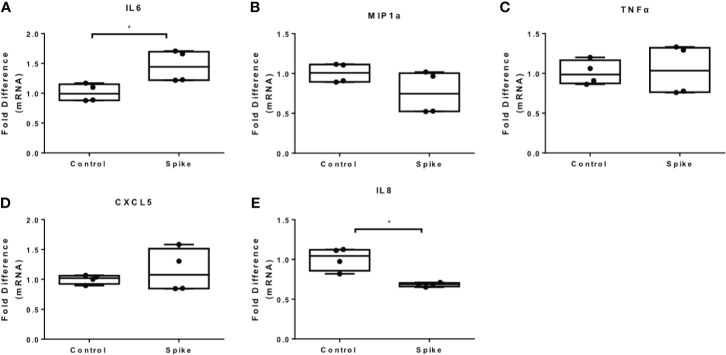
SARS-CoV-2 S protein increased IL6 expression in PAM3csk4-induced THP-1 macrophages. THP-1 macrophages were treated with 20ng/ml SARS-CoV-2 S protein and PAM3csk4 for 12 hours. **(A)** PAM3csk4-induced IL6 expression increased in the presence of SARS-CoV-2 S protein, **(B–D)** No effect observed for MIP1a, TNFα and CXCL5 expression, **(E)** PAM3csk4-induced IL8 expression decreased. Data are illustrated in box-and-whiskers and plotted as median (± min, max). Statistical analysis was performed with Mann – Whitney U test. *p < 0.05.

To determine the effect of SARS-CoV-2 S protein on activated macrophages, we exposed THP-1 macrophages to the TLR4 ligand LPS in the presence of SARS-CoV-2 Spike. LPS-induced IL6 and MIP1a expression was augmented in the presence of SARS-CoV-2 S protein (**Figures 2A, B**), while LPS-induced TNFα was suppressed (**Figure 2C**). Expression of IL8 was not affected (**Figure 2D**) while expression of CXCL5 was moderately reduced at a low concentration of SARS-CoV-2 S protein (**Figure 2E**). We further explored the effect of SARS-CoV-2 S protein on TLR2-activated macrophages using PAM3csk4 as a stimulus. PAM3csk4-stimulated THP-1 macrophages expressed more IL6 in the presence of SARS-CoV-2 S protein (**Figure 3A**), while MIP1a, TNFα and CXCL5 mRNAs were not affected (**Figures 3B–D**). PAM3csk4-induced IL8 mRNA expression was reduced in the presence of SARS-CoV-2 spike (**Figure 3E**).

**Figure 4:**
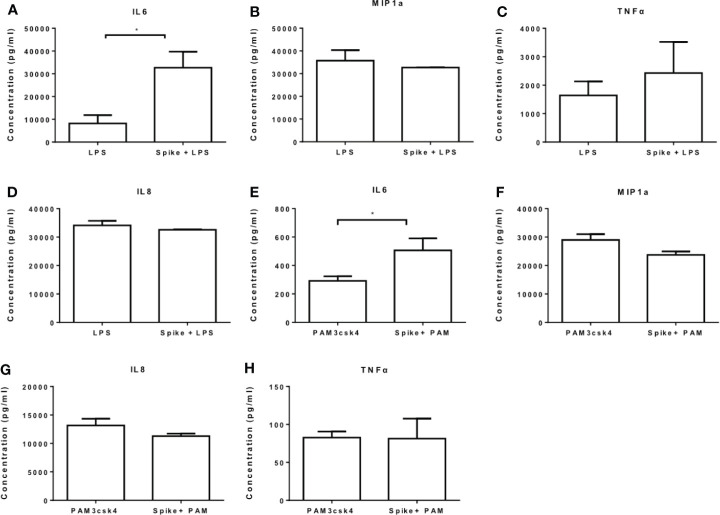
SARS-CoV-2 S protein increased secretion of LPS and PAM3csk4-induced IL6. THP-1 macrophages were treated with 50ng/ml SARS-CoV-2 S protein and LPS or PAM3csk4 for 12 hours and their supernatants were collected for protein quantification using ELISA. **(A)** Increased LPS-induced IL6 in the presence SARS-CoV-2 S protein, **(B–D)** No effect observed for LPS-induced MIP1a, TNFα and IL8 secretion, **(E)** Increased PAM3csk4-induced IL6 secretion, **(F–H)** No effect observed for PAM3csk4-induced MIP1a, IL8 and TNFα secretion. Data are illustrated in bars and plotted as median with range. Statistical analysis was performed with Mann – Whitney U test. *p < 0.05.

We further measured the levels of IL6, MIP1a, TNFα and IL8 at the protein level and confirmed that LPS-induced and PAM3csk4-induced IL6 secretion were augmented in the presence of SARS-CoV-2 S protein, while no effect was observed for the remaining cytokines and chemokines (**Figures 4A–H**). In TLR activated cells the active concentration of SARS-CoV-2 S protein differed from that in naïve cells, which may be due to changes on macrophage sensitivity or ACE2 levels in the presence of TLR ligands.

To assess whether pre-exposure of THP1 macrophages to SARS-CoV-2 S protein altered their response to subsequent LPS or PAM3csk4 stimulations, cells were pre-treated for 6 hours with S protein and then stimulated with the corresponding TLR ligand. The results showed that pre-treatment with S protein resulted in reduced LPS-induced IL6, MIP1a and TNFα expression, as well as reduced IL-6 secretion (**Supplementary Figure 2**), but it did not affect PAM3csk4-induced IL-6, MIP1a or TNFα, suggesting that pre-exposure to S protein, potentially through induction of inflammatory cytokines, triggered events that differentially affected responses to TLR stimuli.

### SARS-CoV-2 S Protein Triggers Pro-Inflammatory Cytokine and Chemokine Expression and Modulates TLR Responses in Primary Human PBMCs and CD14+ Monocytes

**Figure 5:**
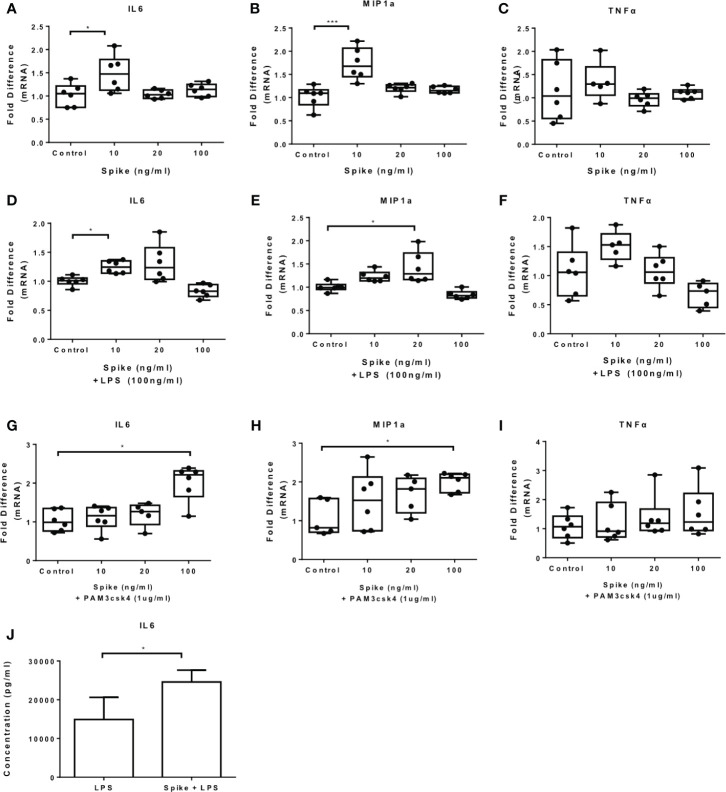
SARS-CoV-2 S protein increased cytokine expression in PBMCs. PBMCs were treated with various concentrations of SARS-CoV-2 S protein and stimulated with TLR ligands. **(A, B)** IL6 and MIP1a expression was increased in the presence of SARS-CoV-2 S protein. **(C)** No effect observed for TNFa expression, **(D, E)** LPS-induced IL6 and MIP1a expression was increased, **(F)** No effect observed for LPS-induced TNFα expression. **(G, H)** PAM3csk4-induced IL6 and MIP1a expression was increased, **(I)** No effect was observed for PAM3csk4-induced TNFα expression, **(J)** Increased IL6 secretion for LPS stimulated PBMCs. Data are illustrated in box-and-whiskers, plotted as median (± min, max) and statistical analysis was performed with Kruskal - Wallis test, regarding qPCR results. For Elisa, data are illustrated in bars, plotted as median with range and statistical analysis was performed with Mann – Whitney U test. *p < 0.05, ***p < 0.001.

To confirm the effect of SARS-CoV-2 S protein on primary human cells, we exposed PBMCs to the S protein at different concentrations and measured the mRNA expression of IL6, MIP1a and TNFα. The results showed that SARS-CoV-2 S protein induced IL6 and MIP1a mRNA and it had no effect on TNFα (**Figures 5A–C**). In the presence of LPS or PAM3csk4 IL6, MIP1a but not TNFα mRNA were induced (**Figures 5D–I**). Only induction of IL-6 was confirmed at the protein level for LPS induced PBMCs (**Figure 5J**). Basal or PAM3csk4-induced cytokines were not detected at protein levels. Induction of IL6 and MIP1a mRNA by SARS-CoV-2 S protein was further confirmed in CD14+ monocytes (**Supplementary Figure 3**).

### Pre-Treatment of THP-1 Macrophages With the ACE2 Receptor Activator Diminazene Aceturate Suppressed SARS-CoV-2 Induced Cytokine Expression

**Figure 6:**
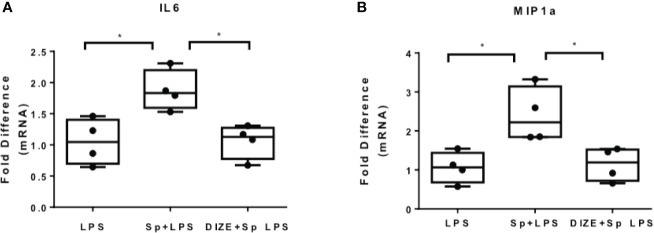
ACE2 activator Diminazene Aceturate (DIZE) reverses the inflammatory status provoked by SARS-CoV-2 S protein. **(A, B)** LPS- induced MIP1a and IL6 expression decreased in THP-1 macrophages pre-treated with DIZE for 6 hours. Data are illustrated in box-and-whiskers and plotted as median (± min, max). Statistical analysis was performed with Kruskal - Wallis test. *p < 0.05.

Since SARS-CoV-2 is internalized with ACE2 receptor, the levels of ACE2 upon infection are reduced. In addition, inhibition of ACE2 enhances inflammation in the context of metabolic disease, while activation of ACE2 suppresses inflammation, suggesting that ACE2 activators may ameliorate the pro-inflammatory action of SARS-CoV-2 S protein (27). In the present study, we used the ACE2 activator DIZE, which enhance the catalytic activity of ACE2 receptor, to determine whether ACE2 activation may suppress the pro-inflammatory action of SARS-Cov-2 S protein. THP-1 macrophages were pre-treated with DIZE for 6 hours and subsequently activated with LPS in the presence of SARS-CoV-2 S protein. Our analysis was focused on LPS-induced IL6 and MIP1a, on which SARS-CoV-2 spike had a prominent effect. The results showed that DIZE reversed the effect of S protein on LPS-induced IL6 and MIP1a (**Figures 6A, B**), suggesting that the pro-inflammatory action of SARS-CoV-2 S protein may be mediated by ACE2 activity.

### SARS-CoV-2 Spike Suppresses IRAK-M Expression in Macrophages

**Figure 7:**
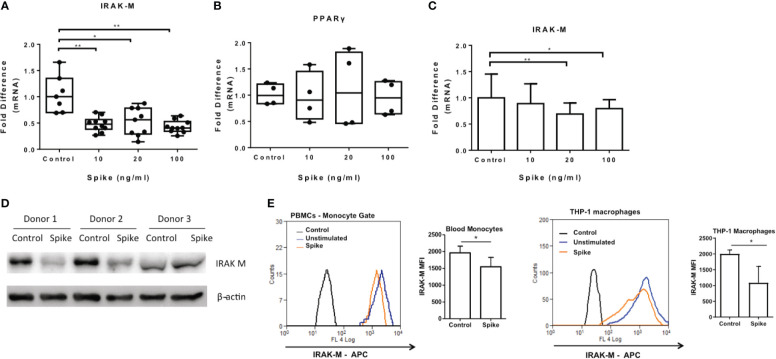
SARS-CoV-2 S protein suppressed IRAK-M expression. THP-1 macrophages, PBMCs and monocytes were treated with different concentrations of SARS-CoV-2 S protein for 12 hours. **(A)** Decreased IRAK-M expression was observed in THP-1 macrophages. **(B)** No effect observed for PPARγ in THP-1 macrophages. **(C)** Decreased IRAK-M expression in PBMCs and CD14+ monocytes respectively was observed. **(D)** Decreased IRAK-M protein levels in western blot from PBMCs of three healthy donors in the presence of SARS-CoV-2 S protein. **(E)** Decreased IRAK-M protein levels in PBMCS and THP-1 macrophages in flow cytometry. Data are illustrated in box-and-whiskers, plotted as median (± min, max) and statistical analysis was performed with Kruskal - Wallis test, regarding THP-1 macrophages **(A, B)**. For PBMCs data are illustrated in bars and plotted as mean ± SD **(C)**. Statistical analysis was performed with One-Way ANOVA test **(C)**. *p < 0.05, **p < 0.01.

We have previously shown that another corona virus, MERS CoV, modulated macrophage responsiveness of macrophages by inducing the expression of IRAK-M and the transcription factor PPARγ, both negative regulators of inflammatory responses. We, therefore examined whether SARS-CoV-2 S protein can affect expression of IRAK-M and PPARγ. The results showed that SARS-CoV-2 S protein suppressed IRAK-M mRNA and protein expression while it had no effect on PPARγ mRNA (**Figures 7A, B**), suggesting that the pro-inflammatory action of SARS-CoV-2 S protein may be mediated by IRAK-M. Similarly, in human PBMCs and CD14+ monocytes IRAK-M mRNA and protein levels were reduced following exposure to SARS-CoV-2 S protein (**Figures 7C–E**).

## Discussion

COVID-19 pandemic is undoubtedly a global health crisis with unprecedented social and economic complications. Most COVID-19 patients have a good prognosis and severe cases usually consist of elderly people or people with underlying diseases and co-morbidities. Severe COVID-19 cases develop acute respiratory distress syndrome (ARDS) with high mortality rates. The severity of COVID-19 is associated with an increased level of inflammatory mediators including cytokines and chemokines and it is characterized as a cytokine release syndrome (CRS) induced by a cytokine storm (21). The excessive inflammation causes multiorgan failure with coagulation abnormalities, cell death, vascular leakage and other complications (28). SARS-CoV-2 infects several cell types including alveolar epithelial cells, alveolar macrophages, monocytes, endothelial cells, all expressing ACE2 receptor and the serine protease TMPRSS2 required for viral entry (7). In the present study, we used THP-1 macrophages stimulated with SARS-CoV-2 S protein as a model to investigate the immunomodulatory action of SARS-CoV-2/ACE2 interaction in macrophages. Our findings demonstrated increased IL6, MIP1a and TNFα expression, suggesting that SARS-CoV-2/ACE2 interaction initiates signals that induce macrophage activation. The concentration of SARS-CoV-2 S protein that triggered the action on macrophages differed between THP1 and primary human cells and the effect on different cytokines was exerted at different concentrations, suggesting a potential role of ACE2 and signalling component expression levels. A recent study demonstrated that SARS-CoV-2 S protein directly induced pro-inflammatory cytokine production, an effect mediated by NFkB and JNK activation and TLR4 signalling (29). These findings are in accordance with other studies supporting that IL6 levels are high in most severe cases and play a crucial role in disease pathogenesis (30). According to different studies, the high severity depends on the cytokine storm that is probably induced by the IL6 amplifier system, a hyper-inflammatory machinery that provokes simultaneous activation of IL6-signal transducer and activator of transcription 3 (STAT3) and NF-κB signalling in non-immune cells (31). This machinery is mediated by the Ang II-AT1R signalling, indicating the implication of SARS-CoV-2/ACE2 interaction in this process, since ACE2 is occupied by SARS-COV-2 resulting to increased serum levels of Ang II (32).

The renin-angiotensin system plays an important role in macrophage activation since AngII promotes inflammatory responses (33), while Ang (1–7) suppresses them (34). Ang (1–7) targets macrophages reducing pro-inflammatory cytokine production including IL6 (35). We, therefore, hypothesize that in the absence of viral infection ACE2 contributes to a local suppression of macrophage responses by reducing AngII and increasing the anti-inflammatory Ang (1–7). In the case of SARS-CoV-2 infection reduction of ACE2 may result in local accumulation of AngII (36). In support of this hypothesis, a recent report demonstrated that glucocorticoids improve severe COVID-19 by activating ACE2 and reducing IL-6 (37). Elevated AngII has been associated with vascular and renal damage and increased production of inflammatory cytokines including IL6 (33), while Ang (1–7) prevents LPS-induced apoptosis of microvascular endothelial cells and development of sepsis (38). Our results provide a potential role for the local RAS system in regulating IL6, known to contribute to COVID-19 pathogenesis.

Since SARS-CoV-2 signals not only *via* ACE2 but also through Toll-like receptors, and macrophages are also exposed to additional TLR ligands during COVID-19, particularly at later stages of COVID-19, we examined whether SARS-CoV-2 S protein can alter the responsiveness of macrophages to TLR signals. For this purpose, we exposed THP-1 macrophages to the TLR4 ligand LPS in the presence of SARS-CoV-2 S protein and found that SARS-CoV-2 S protein augmented LPS-induced IL6 and MIP1a expression. We further explored the effect of SARS-CoV-2 S protein on TLR2-activated macrophages using PAM3csk4 as a stimulus and found that PAM3csk4-stimulated THP-1 macrophages expressed more IL6 in the presence of SARS-CoV-2 S protein. Thus, SARS-CoV-2 augments the inflammatory responses of macrophages triggered through TLR4 or TLR2 stimulation. These findings were further supported by the finding that SARS-CoV-2 S protein decreased IRAK-M expression. IRAK-M, an inactive IRAK kinase, is a negative regulator of TLR signaling, controlling the magnitude of the inflammatory responses of macrophages to TLR signals (39–41). Decreased IRAK-M expression in response to SARS-CoV-2 S protein, implies that the virus modulates TLR signaling, rendering macrophages hyper-responsive to TLR ligands and leading to the hyper-inflammatory state of the COVID-19 disease. There are several studies supporting the participation of TLR signaling and especially TLR4 in the pathogenesis of COVID-19 (42, 43). One study reported upregulation of TLR4 and its downstream signaling mediators in COVID-19 patients (42). In addition, there is evidence supporting that SARS-CoV-2 binds to TLR4 and activates TLR4 signaling to increase cell surface expression of ACE2 facilitating viral entry (43–45). Specifically, it has been shown that TLR4 has the strongest protein-protein interaction with SARS-CoV-2 S protein (43), and that SARS-CoV-2 induces interferon-stimulated gene (ISG) expression by TLR signaling (44), which results in increased expression of ACE2 (45). In addition, increased TLR signaling may contribute to the SARS-CoV-2 mediated lung injury and inflammation since Damage-associated molecular patterns (DAMP)s released from damaged cells also signal *via* TLRs.

We have previously shown that MERS-CoV corona virus induces IRAK-M expression rendering macrophages tolerant and incapable of eliminating secondary infections (25). The present study showed that SARS-CoV-2 had the opposite effect from MERS-CoV, which may explain the fact that COVID-19 is characterized by cytokine storm frequently occurring at early stages of infection, in contrast to MERS-CoV that was associated with immunosuppression of infected individuals (46). Even though the mechanism of IRAK-M suppression by SARS-CoV-2 remains unclear, given the fact that IRAK-M was suppressed at the transcriptional level, a transcriptional or epigenetic mechanism may be involved. Earlier work from us and others has shown that IRAK-M is transcriptionally regulated primarily by c/EBPb (39) and AP1 (47). We, therefore, measured protein levels of c/EBPb in SARS-CoV-2 S protein-treated macrophages and found no effect (data not shown). Expression of IRAK-M is also epigenetically controlled by the PRC complex. The histone demethylase UTX positively regulates IRAK-M while the methyl transferase EZH2 negatively regulates its expression (39). We, thus, hypothesize that SARS CoV2/ACE2 interaction may suppress c/EBPb and/or promote EZH2 expression and subsequently suppress IRAK-M transcription. A recent report showed that SARS-CoV-2 signaling was mediated by IRAK4, indirectly suggesting that the negative regulator of IRAK4 signaling IRAK-M may be inhibited (48).

In the present study, we also demonstrated that the pro-inflammatory action of SARS-CoV-2 S protein may be partly mediated by ACE2 blockade. Engagement of SARS-CoV-2 S protein with ACE2 results in internalization of ACE2 and suppression of its anti-inflammatory activity (17, 18). Enzymatically active ACE2 results in suppression of AngII and upregulation of Ang (1–7), the latter known to possess anti-inflammatory activity (34). We, therefore, hypothesize that treatment with DIZE did not inhibit S protein/ACE2 binding, but rather enhanced ACE2 activity and local production of the anti-inflammatory Ang (1–7), that is otherwise reduced by S protein/ACE2 binding. Thus, the proposed mechanism of action of DIZE on S protein-induced cytokine production is indirect. Our findings showed that activation of ACE2 with the ACE2 activator DIZE ameliorated the pro-inflammatory action of SARS-CoV2 S protein. Consequently, activation of ACE2 could reverse the hyper-inflammatory state led by SARS-CoV-2. DIZE (Berenil) is originally an anti-trypanosome agent for livestock, but it has been proven to reduce pro-inflammatory cytokine (IL6, IL-12 and TNFα) production in macrophages *in vivo* and *in vitro* following stimulation with LPS (26). Several studies, have shown that DIZE enhances the catalytic activity of ACE2, leading to the cleavage of the angiotensin II (49). Animal studies with pulmonary hypertension proved that chronic administration of Diminazene prevented the development of the condition and there was an increased expression of ACE2 mRNA (50). Our results demonstrated not only that ACE2 may mediate the pro-inflammatory action of the virus, but also propose a potential therapeutic approach involving ACE2 activation, which may suppress development of cytokine storm without affecting the immune system capacity, as is the case with current treatments, such as anti-IL6 (Tocilizumab) or corticosteroids (51, 52).

In summary, our study demonstrated that SARS-CoV-2 promoted inflammatory cytokine expression and suppressed IRAK-M, rendering macrophages prone to increased responsiveness to TLR signals, supporting the development of cytokine storm observed in COVID-19 patients. Thus, IRAK-M expression in macrophages may provide a potential biomarker predicting responsiveness of macrophages to infection and development of cytokine storm. Moreover, our findings may propose the use of Diminazene aceturate as a potential treatment for patients with COVID-19, since it is may reverse the pro-inflammatory actions of the virus.

## Data Availability Statement

The raw data supporting the conclusions of this article will be made available by the authors, without undue reservation.

## Author Contributions

CT, AA, EV, and GS designed the study, IP performed experiments, IP, EV, FA and HA, SM analysed data, IP, CT, AAA, EV, and GS drafted the manuscript, IP, CT, AA, EV, FA, HA, SM and GS reviewed the manuscript. All authors contributed to the article and approved the submitted version.

## Funding

This work has been funded by the Hellenic Foundation for Research and Innovation grant (HFRI, General Secretariat for Research and Technology, GSRT Grant No 1010), the King Abdullah International Medical Research Center under grant number RC17/128/R, and the King Faisal Specialist Hospital and Research Center, Riyadh, Saudi Arabia. 

## Conflict of Interest

The authors declare that the research was conducted in the absence of any commercial or financial relationships that could be construed as a potential conflict of interest.
