# Supplementary material for: SARS-CoV-2/ACE2 Interaction Suppresses IRAK-M Expression and Promotes Pro-Inflammatory Cytokine Production in Macrophages
Source: Front Immunol. 2021 Jun 23;12:683800. doi: 10.3389/fimmu.2021.683800 (PMC8261299; doi:10.3389/fimmu.2021.683800)
Supplement: Supplementary file 1 [file DataSheet_1.pdf]

SARS-CoV-2/ACE2 interaction suppresses IRAK-M expression and promotes pro-inflammatory cytokine production in macrophages

Ioanna Pantazi[1,2,*], Ahmed A Al-Qahtani[3,4,*], Fatimah S Alhamlan[3,4], Hani Alothaid[5], Sabine Matou-Nasri[6], George Sourvinos[7], Eleni Vergadi[2] and Christos Tsatsanis[1,8]

[1]Laboratory of Clinical Chemistry and [2]Department of Pediatrics, Medical School, University of Crete, Heraklion 71003, Greece; [3] Department of Infection and Immunity, Research Center, King Faisal Specialist Hospital and Research Center, Saudi Arabia, [4]Department of Microbiology and Immunology, College of Medicine, Alfaisal University, Riyadh, Saudi Arabia; [5]Department of Basic Sciences, Faculty of Applied Medical Sciences, Al-Baha University, Al-Baha, Saudi Arabia; [6]Cell and Gene Therapy Group, Medical Genomics Research Department, King Abdullah International Medical Research Center, Riyadh, Saudi Arabia; [7]Laboratory of Virology, Medical School, University of Crete, Heraklion 70013, Greece; [8]Institute of Molecular Biology and Biotechnology, FORTH, 71100 Heraklion, Greece

Supplementary figures

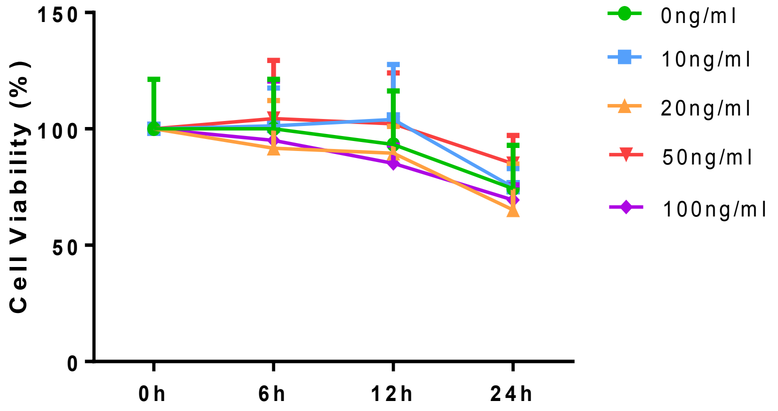

Supplementary Figure S1. Cell viability of THP-1 macrophages stimulated with SARS-CoV-2 S protein. THP-1 macrophages were treated with different SARS-CoV-2 S protein concentrations for 6, 12 and 24 hours and cell viability was assessed with MTT assay. No statistical significant differences were observed among spike protein concentrations at the different timepoints. The results were normalized with the unexposed control. Data are illustrated as line plots with mean ± SD. Statistical analysis was performed with two-way ANOVA. * $p<0.05$

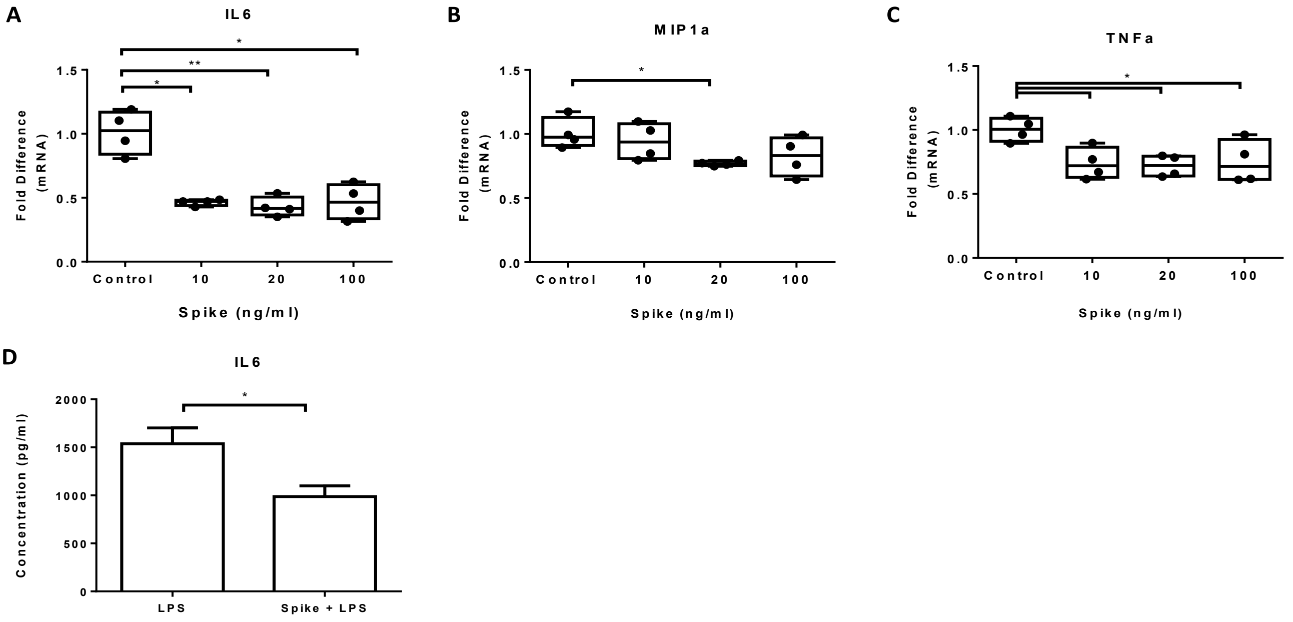

Supplementary Figure 2. Pre-treatment of SARS-CoV-2 S protein decreased cytokine expression in LPS-induced THP-1 macrophages. THP-1 macrophages were pre-treated with SARS-CoV-2 S protein for 6 hours before stimulation with LPS for 12 hours. **A, B. C.** IL6, MIP1a and TNFa expression decreased significantly, **D.** Decreased IL6 secretion. Data are illustrated in box-and-whiskers, plotted as median (± min, max) and statistical analysis was performed with Kruskal - Wallis test regarding qPCR results. For Elisa, data are illustrated in bars, plotted as median with range and statistical analysis was performed with Mann – Whitney U test. *p < 0.05, **p < 0.01

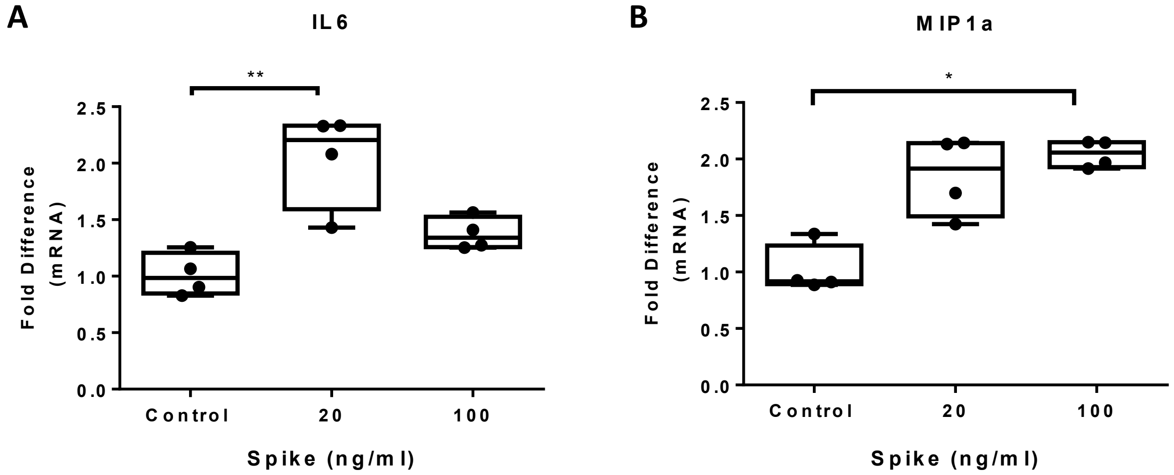

Supplementary Figure 3. SARS-CoV-2 S protein increased cytokine expression in CD14+ monocytes. Monocytes were treated with SARS-CoV-2 S protein (20, 100 ng/ml). **A. B.** IL6 and MIP1a expression was increased in the presence of SARS-CoV-2 S protein. Data are illustrated in box-and-whiskers and plotted as median (± min, max). Statistical analysis was performed with Kruskal - Wallis test. *p < 0.05
